# Slow Freezing Versus Vitrification of Mouse Ovaries: from *Ex Vivo* Analyses to Successful Pregnancies after Auto-Transplantation

**DOI:** 10.1038/s41598-019-56182-8

**Published:** 2019-12-23

**Authors:** Carmen Terren, Maïté Fransolet, Marie Ancion, Michelle Nisolle, Carine Munaut

**Affiliations:** 1 Laboratory of Tumor and Development Biology, GIGA-Cancer, University of Liège, Tour de Pathologie (B23), Sart Tilman, B-4000 Liège, Belgium; 2 Department of Obstetrics and Gynecology, Hôpital de la Citadelle, University of Liège, B-4000 Liège, Belgium

**Keywords:** Mouse, Preclinical research, Molecular medicine

## Abstract

Slow freezing (SF) is the reference method for ovarian tissue cryopreservation. Vitrification (VT) constitutes an alternative but controversial method. This study compares SF and VT (open [VTo] and closed [VTc] systems) in terms of freezing damage and fertility restoration ability. *In vitro* analyses of C57Bl/6 SF or VTo-ovaries, immediately after thawing/warming or after culture (cult), revealed that event though follicular density was similar between all groups, nuclear density was decreased in VTo-ovaries compared to CT-ovaries (CT = 0.50 ± 0.012, SF = 0.41 ± 0.03 and VTo = 0.29 ± 0.044, p < 0.01). Apoptosis was higher in VTo-cult ovaries compared to SF-cult ovaries (p < 0.001) whereas follicular *Bmp15* and *Amh* gene expression levels were decreased in the ovaries after culture, mostly after VTo (p < 0.001). Natural mating after auto-transplantation of SF, VTo and VTc-ovaries revealed that most mice recovered their oestrous cycle. Fertility was only restored with SF and VTo ovaries (SF: 68%; VTo: 63%; VTc: 0%; p < 0.001). Mice auto-transplanted with SF and VTo-ovaries achieved the highest number of pregnancies. In conclusion, *in vitro*, no differences between SF and VTo were evident immediately after thawing/warming but VTo ovaries displayed alterations in apoptosis and follicular specific proteins after culture. *In vivo*, SF and VTo ovary auto-transplantation fully restored fertility whereas with VTc-ovary auto-transplantation no pregnancies were achieved.

## Introduction

In recent decades, considerable therapeutic advances have allowed notable improvements in the survival of cancer patients. It becomes imperative to consider the long-term quality of life of survivors. One critical issue among reproductive-aged cancer survivors is the ability to have biological children. Unfortunately, cancer therapies (radiotherapy and/or chemotherapy) increase the risk of infertility and ovarian failure in very young children. Similar concerns have been raised by young adult women who underwent identical treatments^1^. Thus, the treatments of these patients can lead to premature menopause and, consequently, infertility. The limited options available for fertility preservation include cryopreservation of ovarian cortical tissue containing immature primordial follicles followed by auto-transplantation (OCTP)^2,3^. More importantly, OCTP is the only option available for prepubertal patients, female patients requiring urgent therapy for aggressive malignancies and those suffering from hormone-sensitive malignancies^4^. Different studies have demonstrated the resumption of ovarian function even after transplantation of the prepubertal ovarian cortex^5–8^. To date, more than 130 live births after OCTP have been reported worldwide, with a pregnancy and live birth rate of 33% and 25%, respectively^4,9,10^.

Currently, slow freezing (SF) is the conventional technique used for cryopreservation of the human cortex^11^. Thawing and auto-transplantation are often performed several years later when the patient is cured of her cancer, when she wants to become pregnant and when ovarian failure is observed. Most live births after ovarian cryopreservation have been achieved after SF. However, two successful deliveries have been reported after vitrification (VT)^12^ and two after VT followed by *in vitro* activation of the human ovarian cortex (IVA-OCTP)^13,14^. VT offers the advantages of time efficiency, with no specific or expensive equipment required and, more importantly, no formation of harmful ice crystals. However, VT requires high concentrations of cryoprotectants compared to SF, which carries a risk for cellular toxicity and osmotic trauma^15–17^. VT has gained wide acceptance as the preferred cryopreservation method for oocytes and embryos^18,19^ and could be a potential alternative to SF for ovarian tissue. Several previous studies with human ovarian cortex tissue have shown that VT has no effect on the induction of either the apoptotic process or the follicular pool and increases the preservation of ovarian stroma after thawing^15,20–22^. However, these encouraging results were mostly obtained after tissue analysis immediately after the thawing/warming process. Only a limited number of studies have evaluated ovarian tissue survival after VT and transplantation^23,24^, indicating that additional analyses still need to be performed to confirm the safety and relevance of the VT method before proposing it in clinical practice. However, the number of studies is limited, and direct comparisons between them are difficult or impossible because the methods, the compositions of the cryopreservation solutions and the downstream analysis are completely different. Recently, Lee *et al*. performed a comparison between SF and VT for human ovarian tissue cryopreservation and transplantation onto the back muscle of ovariectomized female SCID mice^25^. Their results indicate that SF is superior to VT in terms of follicle survival and growth after transplantation.

Another critical concern is the biosafety issues of cryopreservation. Currently, procedures are categorized into two types, namely, “open” and “closed” systems, depending on whether there is direct contact with liquid nitrogen (LN_2_). While the open system enables extremely high cooling rates, the direct contact with LN_2_ increases potential cross-contamination risks^26,27^. To address these safety considerations, closed systems were developed, first and mainly for oocyte and embryo cryopreservation^28,29^, and are currently the only ones used in laboratories.

The first aim of our study was to investigate the effect of the cryopreservation method (SF and VT) on the ability of murine ovaries to support folliculogenesis after thawing/warming. Possible cryoinjuries were assessed by performing histological, immunohistochemical and molecular analyses of control (CT), SF and VTo ovaries before and after *in vitro* culture. Apoptosis, cell proliferation, follicular density and the expression of a variety of genes specific to follicular integrity, health, and viability were evaluated.

The second aim was to analyse the resumption of ovarian and reproductive functions *in vivo* after cryopreservation and auto-transplantation of ovaries into mice with ovarian failure.

## Materials and Methods

### Collection and preparation of ovaries for *in vitro* studies

C57Bl/6 mice (*n* = 82, 4 weeks old) were obtained from Charles River Laboratories (USA). The ovaries were removed through small dorsolateral skin incisions and were placed either in Leibovitz L-15 medium (Lonza, Verviers, Belgium) supplemented with 10% Fetal Bovine Serum (FBS; Thermo Fisher Scientific, Gibco, Waltham, Mass., USA) (transport solution for slow freezing (SF)) or in Dulbecco’s phosphate-buffered solution (DPBS; Thermo Fisher Scientific, Gibco, Waltham, Mass., USA) supplemented with 20% FBS (transport solution for vitrification (VT)). Leibovitz L-15 is the conventional medium for the SF procedure, whereas there is no consensus for ovarian tissue VT. Therefore, we used Youm’s VT procedure^30^. In order to cryopreserve only the ovary, adjacent tissues taken at the time of ovariectomy were removed under a binocular using a scalpel. The Animal Ethics Committee of the University of Liège approved this study (# 1547) and all experiments were performed in accordance with relevant guidelines and regulations.

### Slow freezing (SF) and thawing procedure

Ovaries were placed in cryopreservative medium containing Leibovitz L-15 medium supplemented with 10% FBS, 10% dimethylsulfoxide (DMSO; Merck, Darmstadt, Germany) and 0.1 M sucrose. After equilibration in cryopreservation medium for 30 min at 4 °C, ovaries were placed in cryovial tubes (Simport, Montreal, Quebec, Canada) and subsequently cooled in a programmable freezer (CL-8800i System; CryoLogic, Mulgrave, Victoria, Australia) as described previously^31^ and stored in liquid nitrogen.

For thawing, cryovials were incubated at room temperature for 2 min and thawed by rapid immersion at 37 °C in a water bath. To remove cryoprotectants, ovaries were washed three times for 5 min at 37 °C in Leibovitz L-15 medium.

### Vitrification (VT) and warming procedure

Ovaries were placed in DPBS supplemented with 20% FBS (transport medium). The vitrification procedure is composed of a two-step protocol with ascending concentrations of ethylene glycol (EG) and DMSO in basic transport solution. Ovaries were first incubated in 7.5% EG and 7.5% DMSO for 10 min then 20% EG, 20% DMSO and 0.5 M sucrose for 5 min as described by Youm *et al*.^30^. All steps of vitrification were carried out on ice^32^ The ovaries were either directly immersed in liquid nitrogen before being transferred in cryotubes (Sarstedt, Nümbrecht, Germany), previously immersed in liquid nitrogen (open method) or placed in a high security freezing straw (closed method) (Cryo Bio System, L’Aigle, France).

For warming, to limit osmotic shock, ovaries were soaked for 5 minutes at 37 °C in solutions with decreasing concentrations of sucrose (1, 0.5, 0.25 and 0 M sucrose in DPBS-20% FBS).

### *In vitro* culture

After thawing/warming, some ovaries were cultured for 4 hours at 37 °C in 96-well plates (see Table S1 for the experimental distribution). Culture medium was composed of Dulbecco’s Modified Eagle Medium (Thermo Fisher Scientific, Gibco, Waltham, MA, USA) supplemented with 10% decomplemented and desteroidized FBS, 200mM L-Glutamine (Thermo Fisher Scientific, Gibco, Waltham, MA, USA), 1% of a mixture of insulin (1000 mg/l), transferrin (550 mg/l) and selenium (0.67 mg/l) (Thermo Fisher Scientific, Gibco, Waltham, MA, USA), 1 μl/ml penicillin-streptomycin (Thermo Fisher Scientific Gibco 15140–122 Waltham, MA, USA), 100 μg/ml ascorbic acid (Sigma-Aldrich, St. Louis, MO, USA) and 0.2% human FSH (Sigma-Aldrich, St. Louis, MO, USA).

### Histological assessment

Ovaries fixed in 4% formaldehyde were paraffin-embedded and serially sectioned (5 µm sections). Haematoxylin and eosin (H&E) staining was used for differential follicle counts. The scanned H&E sections were analysed using NDP view software (NDP.view2 Viewing software U12388-01, Hamamatsu Photonics K.K., Japan). Sections were analyzed by light microscopy for the presence of primordial, primary, secondary and antral follicles based on morphological classification of mouse follicles^33^. The follicular densities (number/mm^2^) were calculated after manually outlining the ovarian surface (NDP view software).

Ovarian viability was evaluated by nucleus count after haematoxylin staining by computer-assisted image analysis. Using Photoshop CS4 software (Adobe Systems Incorporated, San Jose, CA, USA), a mask was created to delineate the area to be analysed. The number of cells labelled per mm^2^ was then quantified using MATLAB 9.0.0.341360 (R2016a) software (MathWorks, Inc.).

Apoptosis and cell proliferation in the ovaries were evidenced by immunostaining of caspase-3 and Ki67, respectively. Sections were deparaffinised and rehydrated, and endogenous peroxidase activity was blocked by incubating the sections in 3% hydrogen peroxide for 20 min at room temperature (RT). Nonspecific binding sites were blocked by incubation with phosphate-buffered saline (PBS) containing 10% bovine serum albumin (BSA) or with serum of the species from which the secondary antibody comes from, for 1 h at RT. Ki67 primary antibody (Abcam, Cambridge, UK) was diluted 1/100 in PBS-BSA 1% and incubated for 1 h at RT. Anti-cleaved caspase-3 antibody (Cell Signaling, Danvers, USA) was diluted 1/300 in the REAL antibody diluent (Dako, Glostrup, Denmark) and incubated overnight at 4 °C, followed by incubation with the secondary antibody HRP linked (ENVISION/HRP ready to use, Dako, Glostrup, Denmark) for 30 min at RT. The reaction was revealed using DAB + (Dako, Glostrup, Denmark) and the sections were counterstained with haematoxylin.

For the quantitative analysis of immunostaining of caspase-3, labelled follicles were manually counted. Follicles were considered to be labelled when there were more than 30% of labelled cells within a follicle.

Cell proliferation was determined by computer-assisted image analysis on Ki67-labelled sections. Using Photoshop CS4 software, a mask was created to delineate the area to be analysed. The number of cells labelled per mm^2^ was then quantified using MATLAB software.

### RT real-time-PCR for mRNA quantification

Total RNA from ovaries was extracted using the RNeasy Mini Kit (Qiagen, Valencia, CA, USA), following the manufacturers protocol. 900 ng of RNA was then reverse transcribed into cDNA using the Transcriptor First Strand cDNA Synthesis Kit (Roche, Basel, Switzerland). Real-time quantitative PCR were performed using specific primers and either Brilliant SYBR GREEN qPCR master mix (Roche, Basel, Switzerland) or FastStart Essential DNA Probes Master (Roche, Basel, Switzerland) and a specific probe (Universal Probe Library, Roche, Basel, Switzerland) on a LightCycler® 480 (Roche, Basel, Switzerland). Primer sequences for the target genes and corresponding probes are summarized in Table 1. Gene expression values were normalized to a housekeeping gene (*Gapdh*), and mRNA expression levels were quantified using the ∆CT method.

**Table 1:** Primer sequences and product size for RT-qPCR analysis.

Gene	Primer sequences	Product size (bp)	UPL Probe (Roche)
*Amh*	F: 5′-GGG-GAG-ACT-GGA-GAA-CAG-C-3′	67	41 (ref: 4688007001)
	R: 5′-AGA-GCT-CGG-GCT-CCC-ATA-3′		
*Bax*	F: 5′-AGT-GTC-TCC-GGC-GAA-TTG-3′	69	56 (ref: 4688538001)
	R: 5′-CCA-CGT-CAG-CAA-TCA-TCC-T-3′		
*Bcl-XL*	F: 5′-TGA-CCA-CCT-AGA-GCC-TTG-GA-3′	78	2 (ref: 4684982001)
	R: 5′-GCT-GCA-TTG-TTC-CCG-TAG-A-3′		
*Becn1*	F: 5′-GAC-TCG-ATT-TTG-TCT-TCC-GTA-CA-3′	94	16 (ref: 4686896001)
	R: 5′-CTG-GGT-TTT-GAT-GGA-ATA-GGA-G-3′		
*Bmp15*	F: 5′-CAG-TAA-GGC-CTC-CCA-GAG-GT-3′	113	21 (ref: 4686942001)
	R: 5′-AAG-TTG-ATG-GCG-GTA-AAC-CA-3′		
*Gdf9*	F: 5′-TTC-GTG-TGT-GCC-GGG-CAA-GT-3′	196	SYBR Green
	R: 5′-GTC-ACA-GGA-AGC-TCT-CTG-CCC-A-3′		
*Gapdh*	F: 5′-TGT-CCG-TCG-TGG-ATC-TGA-C-3′	151	80 (ref: 4689038001)
	R: 5′-GAG-TTG-CTG-TTG-AAG-TCG-CA-3′		

### Western blot analysis

Two ovaries were pooled by condition to obtain a sufficient protein concentration. The protein extraction was carried out with 175 μl of radio immunoprecipitation assay (RIPA) buffer containing 4% of a protease inhibitor (Roche, Basel, Switzerland). Lysate were collected and protein concentrations determined using a protein assay kit (Bio-Rad Laboratories, Hercules, CA, USA). Equal amounts of protein were denatured and separated by electrophoresis on 15% SDS-polyacrylamide gels and then transferred onto a polyvinylidene difluoride membrane (PerkinElmer, Waltham, MA, USA) at 100 V for 1 h. After blocking, proteins were incubated with respective primary antibodies in blocking solution according to the manufacturer’s protocol (Table 2). The appropriate horseradish peroxidase-conjugated secondary antibody was added to the membrane followed by a 1 h incubation at RT. After sequential washing of the membranes to remove excess secondary antibody, signals were detected using an enhanced chemiluminescence (ECL) kit (PerkinElmer, Waltham, MA, USA) according to the manufacturer’s instructions in a LAS4000 imager (Fujifilm, Tokyo, Japan).

**Table 2:** Antibodies used for Western blot analysis.

Target Protein	Blocking Solution	Primary Antibody	Secondary Antibody
BMP15	Milk 5%	Rabbit monoclonal antibody (Abcam, Cambridge, UK) 1:2000	Goat anti-rabbit HRP-linked antibody (Cell signalling, Danvers, USA) 1:2000
CASPASE-3	Milk 5%	Rabbit monoclonal antibody (Cell signalling, Danvers, USA) 1:1000	Goat anti-rabbit HRP-linked antibody (Cell signalling, Danvers, USA) 1:2000
LC3	Casein	Mouse Monoclonal antibody (Nanotools, Teningen, Germany) 1:1000	Horse anti-mouse HRP-linked antibody (Cell signalling, Danvers, USA) 1:2000

### Mice model of induced ovarian failure

A unilateral ovariectomy was performed on 8-week-old C57Bl/6 mice. After one week, mice were daily intraperitoneally treated with vinylcyclohexene dioxide (VCD, Sigma-Aldrich, St. Louis, MO, USA) for 19 days (100 μl/mouse at 160 mg/kg of VCD diluted in sesame oil) to induce ovarian failure^34^. Mice in the control group (n = 5) were treated with the vehicle. Two months after the last VCD injection, the status of oestrous cycle was determined by daily vaginal cytology^35^. The onset and cessation of cyclicity were determined by the cell population in the vaginal lavage. Mice were considered to be in menopause after 2 weeks of consecutive dioestrous. Mice in the control group kept their cyclicity (Fig. S2).

### Auto-transplantation

After confirmation of menopause, cryopreserved-thawed/warmed ovaries were auto-transplanted. Briefly, the remaining non-functional ovary was externalized under gas anaesthesia (Isoflurane, Dechra, Northwich, UK). Under a binocular, using thin spring scissors, a small incision was made at the level of the ovarian bursa. The frozen-thawed/warmed ovary was then slipped through this incision into the ovarian bursa that still contains the non-functional ovary. The ovaries were then gently placed back into the peritoneal cavity before closing the peritoneum and the skin. Sham operation was performed in the control group.

After the auto-transplantation, daily vaginal smears were performed to detect a possible resumption of ovarian cycles. The auto-transplanted female mice which had recovered an estrous cycle after surgery as well as control mice (unilaterally ovariectomized and sham operated) were paired with male C57Bl/6 with proven mating abilities (one male for 2–3 females). Once pregnancy was confirmed either by visualization of a vaginal plug or by increasing weight, the females were housed individually. If no vaginal plug was detected, pairing was continued up to 18 weeks. The number of females that gave birth and of pups born alive was recorded. The experimental design is reported in a flow chart (Supplemental, Fig. S2).

### Statistical analysis

Statistical analyses were performed using GraphPad Prism software (GraphPad, San Diego, CA, USA). All data are presented as means ± SEM. Nonparametric Kruskal-Wallis test followed by multiple comparison Dunn’s post-test was applied for multigroup comparisons. The comparison between the two freezing techniques on the fecundity of females after auto-transplantation and natural mating was carried out by Chi-Square statistical test. A probability of p < 0.05 was considered to be statistically significant.

## Results

### *In vitro* study: Influence of the cryopreservation method

#### Histological evaluation and follicular counts

**Figure 1:**
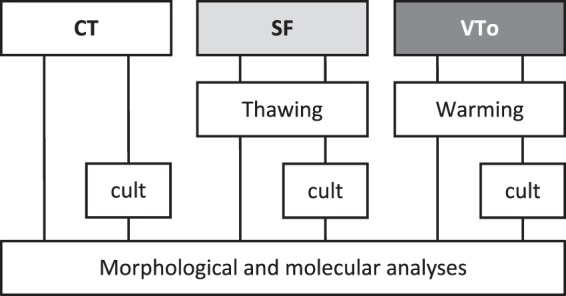
Experimental design for *in vitro* analysis. Morphological and molecular analyses of follicular density, apoptosis and follicular specific genes expression (immunohistochemistry, western-blot, RT-qPCR) in slow frozen/thawed and vitrified/warmed ovaries were done in parallel and compared to fresh control ovaries. CT: control/fresh ovaries; SF: slow frozen/thawed ovaries, VTo: open vitrified/warmed ovaries, cult: *ex vivo* culture during 4 hours.

**Figure 2:**
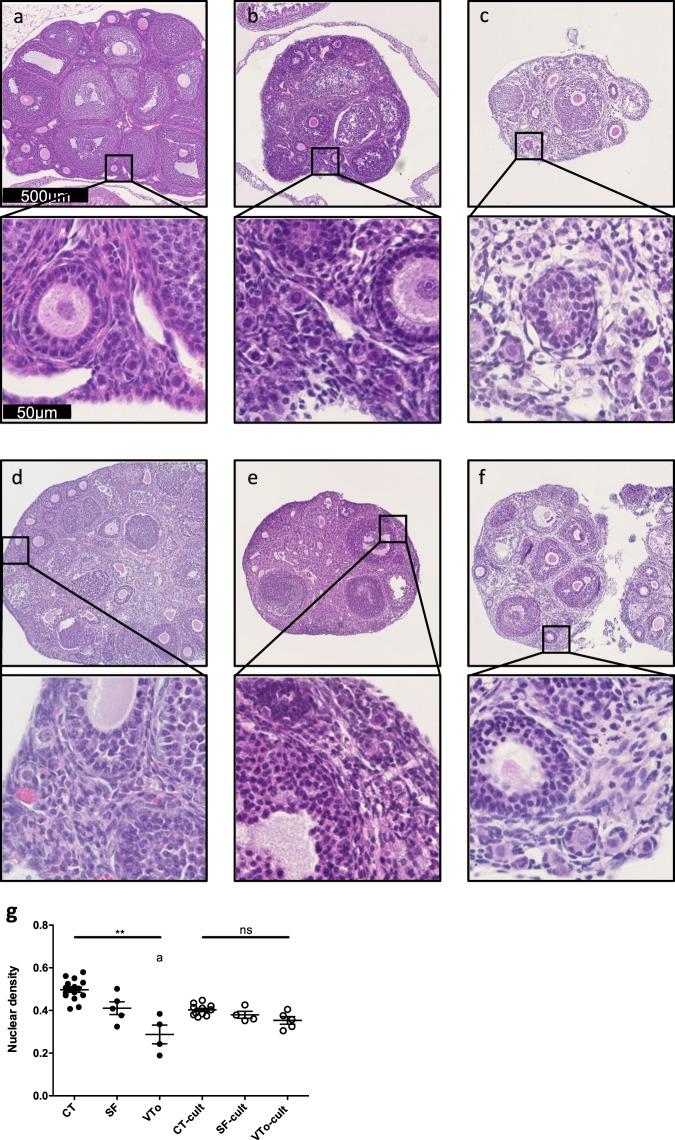
Morphological analysis. Representative images of the haematoxylin & eosin staining of ovaries from the different groups before (**a–c**) and after 4 h of culture (**d–f**). Fresh-(CT) (**a,d**), slow frozen/thawed (SF) (**b,e**) and open vitrified/warmed ovaries (VTo) (**c**,**f**). (**g**) Computer-assisted quantification of nuclear density (mean ± SEM). n = 4–16 ovaries per group. ns: non-significant, ** p ≤ 0.01, ^a^different from CT.

**Figure 3:**
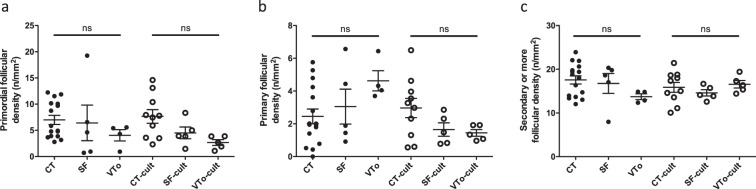
Follicular density analysis. Number/square millimetre of primordial (**a**), primary (**b**) or secondary or more follicles (**c**) (mean ± SEM). n = 4–16 ovaries per group. CT: control/fresh ovaries; SF: slow frozen/thawed ovaries, VTo: open vitrified/warmed ovaries, cult: *ex vivo* culture during 4 hours. ns: non-significant.

The experimental design is summarized in Fig. 1. Morphological analyses of the ovaries revealed that the morphology of SF ovaries was similar to that of CT ovaries. Follicular integrity and stromal structures were better preserved after SF than after VTo (Fig. 2). The stromal density decreased in both cryopreserved groups of ovaries. The density of the granulosa cells associated with growing follicles was higher after SF than after VTo (Fig. 2b,c). However, in all groups, the primordial follicles displayed ]a healthy appearance (Fig. 2a–c). After 4 h in culture, the prevalence of stromal cell damage increased in all groups, accompanied by cell losses, with VTo ovaries being the most affected (Fig. 2d–f). The nuclear density evaluated after haematoxylin staining and computer-assisted quantification was decreased in VTo ovaries compared to CT ovaries (CT = 0.50 ± 0.012, SF = 0.41 ± 0.03 and VTo = 0.29 ± 0.044, mean ± SEM, p < 0.01). After 4 h of culture, there were no additional differences between the groups (Fig. 2g). The follicular count according to follicle maturation was evaluated next. There were no differences in the follicular densities of the primordial follicles between groups either after thawing/warming or after 4 h of culture (Fig. 3a). The mean primary or growing follicle density was similar between all groups (Fig. 3b,c).

#### Cell proliferation

**Figure 4:**
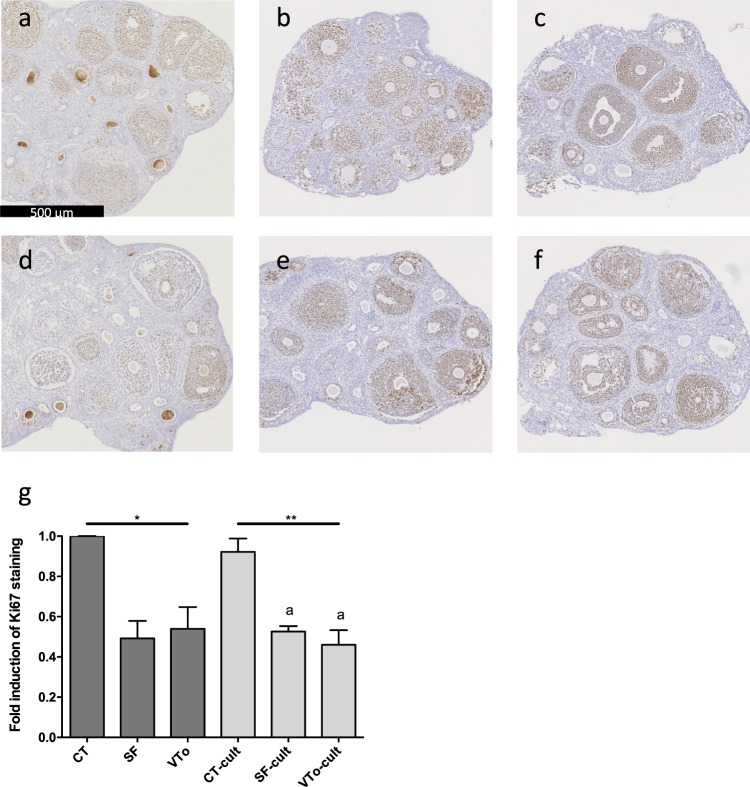
Proliferation analysis with representative illustration of Ki67 immunostaining for CT (**a,d**), SF (**b,e**) and VTo (**c,f**) ovaries cultured (**d–f**) or not (**a–c**) for 4 hours. (**g**) Computer-assisted quantification of Ki67 immunostaining (mean ± SEM). n = 4–16 ovaries per group. CT: control/fresh ovaries; SF: slow frozen/thawed ovaries, VTo: open vitrified/warmed ovaries, cult: *ex vivo* culture during 4 hours. *p ≤ 0.05, **p ≤ 0.01, ^a^different from CT-cult.

The health and proliferative status of the stroma and granulosa cells were assessed by Ki67 immunostaining of ovarian sections. As expected, in the CT ovaries, the immunolabelling of the granulosa cells was strong, while the stroma was totally devoid of Ki67 staining (Fig. 4a–f). After cryopreservation and *in vitro* culture, proliferation decreased in all cases compared to that in the CT or CT-cult ovaries (Fig. 4g).

#### Follicular integrity and functionality

**Figure 5:**
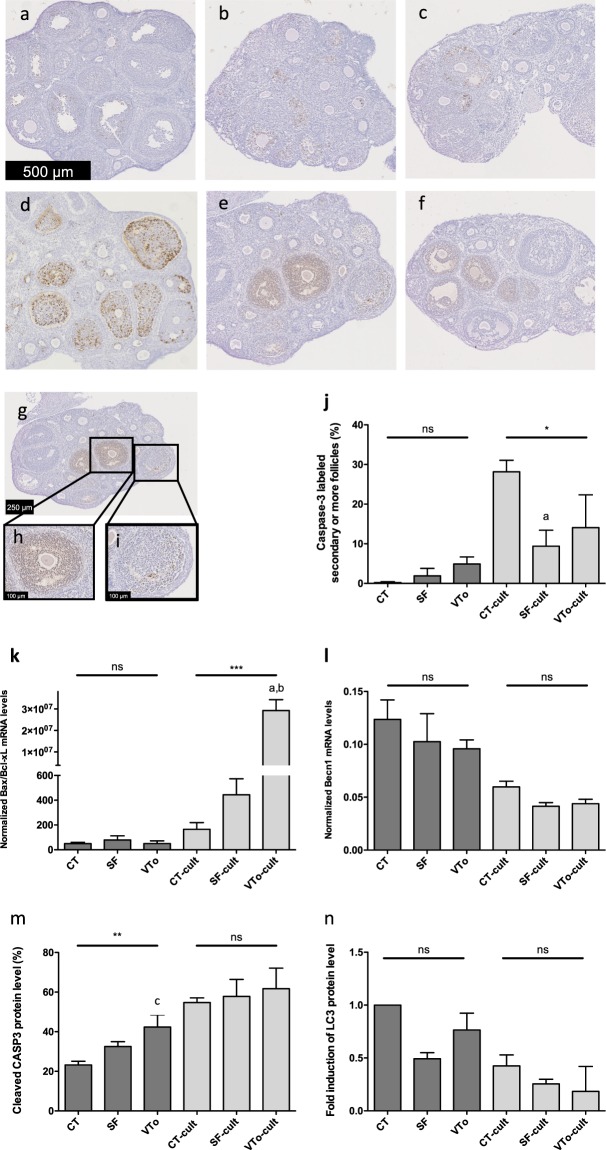
Molecular analysis with representative images of active caspase-3 immunostaining of CT (**a,d**), SF (**b,e**) and VTo (**c,f**) ovaries cultured (**d–f**) or not (**a–c**) for 4 hours. Illustration of labelled antral follicles (**g**) with more (**h**) or less (**i**) than 30% of active caspase-3 positive granulosa cells. (**j**) Computer-assisted quantification of active caspase-3 immunostaining. (**k**) Bax/Bcl-xl mRNA ratios and (**l**) Becn1 mRNA levels in the different groups. (**m**) Western blot quantification of cleaved caspase-3 and (**n**) LC3 protein level in the different groups. Results are expressed as means ± SEM. n = 4–16 for (**a–j**); n = 9–22 for (**k,l**); n = 5–16 for m and n per group. CT: control/fresh ovaries; SF: slow frozen/thawed ovaries, VTo: vitrified/warmed ovaries, cult: *ex vivo* culture during 4 hours. ns: non-significant, *p ≤ 0.05, **p ≤ 0.01, ***p ≤ 0.001, ^a^different from CT-cult, ^b^different from SF-cult and ^c^different from CT.

To assess apoptosis, immunohistochemistry for active caspase-3 (CASP3) was performed on ovarian sections. In all sections, active caspase-3 was only found in secondary or more mature follicles (Fig. 5a–f). In CT ovaries, almost no staining was observed (Fig. 5a). After quantification (Fig. 5g) of follicles with a staining coverage of more (Fig. 5h) or less (Fig. 5i) than 30%, a trend towards an increase in cell death was observed after both cryopreservation methods (1.9 ± 1.9 in SF, 4.9 ± 1.8 in VTo versus 0.2 ± 0.2 in CT, mean ± SEM) (Fig. 5j). A further increase in active CASP3 was observed in all groups after 4 h of *in vitro* culture, with the CT ovaries being the most affected (Fig. 5d–f). The SF-cult ovaries displayed a lower level of active caspase-3 than the CT-cult ovaries (9.4 ± 4.0 in SF-cult, 14 ± 8.3 in VTo-cult versus 28.2 ± 2.9 in CT-cult, mean ± SEM, p < 0.05) (Fig. 5j). Western blot analysis confirmed the increase in cleaved caspase-3 after cryopreservation (p < 0.01, Figs. 5m and S1a). RT-qPCR analysis was performed to evaluate the *Bax/Bcl-xl* mRNA ratios to assess susceptibility to apoptosis and to evaluate *Becn1* mRNA levels as a marker of autophagy. Cryopreservation by SF or VTo did not affect the *Bax/Bcl-xl* ratio or the *Becn1* mRNA level in the different groups of ovaries. After 4 h in culture, the *Bax/Bcl-xl* ratios increased in all groups, with VTo-cult ovaries being the most affected (p < 0.001, Fig. 5k). Conversely, a tendency towards a decrease in *Becn1* mRNA was observed in all groups of cultured ovaries compared to the results obtained after cryopreservation (Fig. 5l). Western blot analysis of LC3 protein also confirmed the decreasing trends in culture in all conditions after 4 h (Figs. 5n and S1b).

**Figure 6:**
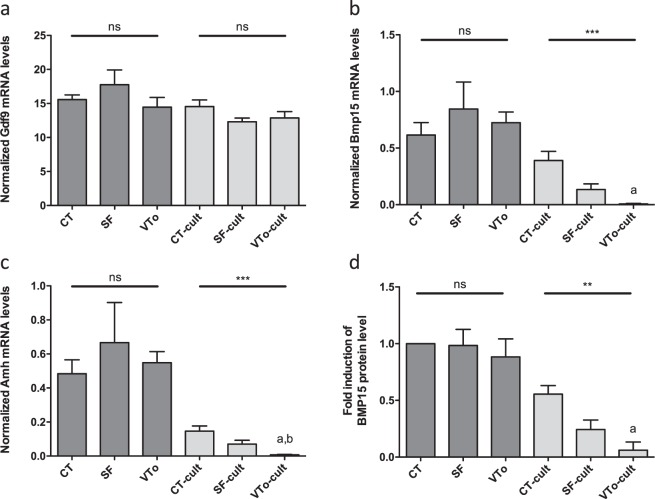
Quantification of the expression level of *Gdf9* (**a**), *Bmp15* (**b**), *Amh* (**c**) mRNA in the different groups. (**d**) Western blot quantification of BMP15 protein in the different groups. Results are expressed as means ± SEM. n = 9–22 per group for a-c; n = 5–16 per group for d. CT: control/fresh ovaries; SF: slow frozen/thawed ovaries, VTo: open vitrified/warmed ovaries, cult: *ex vivo* culture during 4 hours. ns: non-significant, **p ≤ 0.01, ***p ≤ 0.001, ^a^different from CT-cult and ^b^different from SF-cult.

RT-qPCR analysis of the mRNA levels of *Gdf9*, a gene required for ovarian folliculogenesis, revealed no difference between SF or VTo ovaries compared to CT ovaries. No further difference in Gdf9 mRNA was observed after *in vitro* culture for 4 h for either cryopreservation method (Fig. 6a). The *Bmp15* and *Amh* mRNA levels were similar between the CT, SF and VTo ovaries. After 4 h in culture, the *Bmp15* and *Amh* mRNA levels decreased in all groups, with the most notable decrease in the VTo-cult ovaries (p < 0.001, Fig. 6b,c). Western blot analysis of BMP15 protein confirmed the RT-qPCR results (Figs. 6d and S1c).

### *In vivo* study: Comparison of the SF and VT methods

The experimental design for this portion of the study is summarized in Fig. S2 (Supplemental data). Mice were randomly assigned to an experimental group, and unilaterally removed ovaries were treated accordingly (CT, n = 5; SF, n = 22; VTo with open system, n = 11; and VTc with closed system, n = 11). Vaginal cytology analysis was performed from the eighth week after the first injection of VCD, and oestrous cyclicity was absent in approximately 75% of all mice. Ovarian failure was complete in all mice within the subsequent 3 weeks (Fig. S3). Auto-transplantation of the cryopreserved ovaries into the remaining bursa of the follicle-depleted non-functional ovary was performed. Vaginal cytology analysis of the auto-transplanted mice showed that one week later, some mice had already recovered their oestrous cyclicity. Most mice resumed their ovarian cycles 3 weeks after auto-transplantation (SF: 95%; VTo: 91%; VTc: 82%) (Table 3). There was no significant difference in the time required for the recovery of the oestrous cycle between the groups of females receiving SF, VTo or VTc ovaries. All females with recovered oestrous cycles were naturally mated, and their fecundity was assessed. Table 3 summarizes the fecundity of the grafted mice examined by natural mating after transplantation (associated statistical analyses are presented as Supplemental data (Table S2)). Twenty weeks after auto-transplantation, all mice in the CT group had become pregnant, and most of them gave birth to viable pups. More than 60% of the mice auto-transplanted with SF or VTo ovaries were pregnant 20 weeks after auto-transplantation and delivered pups. In contrast, mice with VTc-auto-transplanted ovaries never became pregnant (p < 0.001).

**Table 3:** Fecundity of females without (CT) or after auto-transplantation with SF or VT ovaries examined after natural mating.

	CT*	SF	VTo	VTc	p value
Number of females	5	22	11	11	NA
Recovered estrous (3 weeks after transplantation)	NA	21 (95%)	10 (91%)	9 (82%)	0.4382^a^
Pregnant mice (20 weeks after transplantation)	5 (100%)	15 (68%)	7 (63%)	0 (0%)^c^	0.0002^a^
Number of pregnancies^d^ (0, 1, 2, 3 = mice that were never or 1, 2 or 3 times pregnant)	0: 0 (0%)	0: 7 (32%)	0: 4 (36%)	0: 11 (100%)	0.0005^a^
	1: 2 (40%)	1: 3 (14%)	1: 6 (55%)	1: 0 (0%)	
	2: 2 (40%)	2: 9 (41%)	2: 1 (9%)	2: 0 (0%)	
	3: 1 (20%)	3: 3 (14%)	3: 0 (0%)	3: 0 (0%)	
Litter size (mean ± SEM)	3.75 ± 0.59	3.7 ± 0.51	2.8 ± 0.86	NA	0.6089^b^
Surviving litters	3/9 (33.3%)	14/30 (47%)	1/8 (12.5%)	NA	0.1983^a^

*Females were unilaterally ovariectomized at the same time than the others, daily treated with vehicle, sham operated at the day of the auto-transplantation and paired with C57Bl/6 males in parallel with auto-transplanted females; NA: not applicable; ^a^Chi-square test; ^b^Kruskal-Wallis test with post hoc analysis (see materials and methods); ^c^VTc versus all groups (p < 0.001); ^d^VTc versus all groups (p < 0.001).

The number of pregnancies among all groups was significantly different (p = 0.0005). In both the CT and SF-ovary auto-transplantation groups, the female mice delivered more than once, with no differences between these 2 groups. The delivery rate was also similar for mice with VTo ovaries than for those with SF ovaries. The VTo mice mostly delivered only once. The mean litter sizes of the CT and SF groups were similar but tended to be lower in the VTo group (CT: 3.75 ± 0.59, SF: 3.7 ± 0.51, VTo: 2.8 ± 0.86, mean ± SEM). However, in all groups, several litters did not survive. This was most apparent in the VTo group, in which only one litter survived. Among pups that were not victims of maternal cannibalism, all showed normal further development.

**Figure 7:**
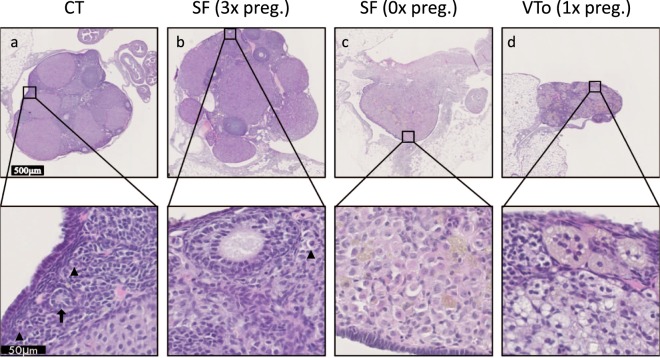
Representative images after H&E staining of ovaries after the sacrifice. (**a**) Control ovary; (**b**) SF-ovary-auto-transplanted mice that gave birth three times; (**c**) SF-ovary-auto-transplanted mice that was never pregnant; (**d**) VTo-ovary-auto-transplanted mice that gave birth once. Arrowheads indicate primordial follicles. Arrow indicates primary follicle.

All females were sacrificed 8 months after unilateral ovariectomy, and ovaries were histologically examined after haematoxylin & eosin staining. Both normal follicular development and corpora lutea formation were observed in the CT group and SF-graft ovary group of previously pregnant mice (Fig. 7a,b), whereas in the VTo graft ovary group, mostly corpora lutea were found (Fig. 7d). When the SF grafts from the mice that never became pregnant were analysed, no more resting or developing follicles were detected (Fig. 7c).

## Discussion

Ovarian tissue cryopreservation aims to increase the chances of prospective fertility in cancer patients after treatment by means of transplantation. Currently, SF is considered the “gold standard” for ovarian tissue cryopreservation, and most of the reported live births after auto-transplantation have been achieved with this method^4^. Additionally, VT has recently been used^36,37^. By itself, cryopreservation of single cells, such as oocytes, is already challenging, but cryopreservation has become more complicated for complex organs composed of heterogeneous cell types, such as the ovary. Indeed, each cell type has its own requirements for preservation. These requirements can be so different among different cell types that it can become impossible to develop an optimized cryopreservation protocol^38,39^.

VT is a simple, effective and widely used method for the cryopreservation of oocytes and embryos, taking the place of traditional SF in assisted reproductive technology^29,40–42^. The associated survival rates of oocytes and embryos after the freezing-thawing procedure are similar to those of SF methods^20^. Although oocyte freezing is now mostly performed with VT, there is still controversy regarding the use of SF or VT for ovarian tissue cryopreservation.

In the first part of our study, cryoinjury damage due to SF versus VT was examined *in vitro*. Immediately after freezing/thawing, the global morphology of the SF ovaries was similar to that of the fresh ovaries, and these ovaries were better preserved than the VTo ovaries. Our results are in agreement with those of Fabbri *et al*.^43^, although several other studies have revealed a better stromal cell ultrastructure after VT than after SF^20,22,44^. The results concerning follicular morphology after cryopreservation are not always consistent. For example, in Isachenko’s study, cryopreservation did not alter follicular morphology^45^, while in several other studies, both SF^46^ and VT had less damage in terms of follicle morphology^44^. Several reasons for such divergent results could be the heterogeneity of follicle abundance and distribution, inconsistent tissue size and the different types and concentrations of cryoprotectants. Furthermore, morphological analyses performed just after cryopreservation and thawing could not truly reflect all the potential damage done by the procedure^47^. Therefore, we analysed and quantified follicles after *in vitro* culture. No differences were observed in the follicular density between groups in agreement with another recent study with ewe ovarian cortex^48^. However, active caspase-3 was also mostly associated with secondary follicles. These results confirm the findings of previous studies on the human ovarian cortex, in which only granulosa cells associated with antral follicles from cryopreserved ovaries (SF and VT) were apoptotic^24^, indicating that growing follicles that are metabolically more active are more sensitive to cryopreservation^17^. By Western blot analysis, the level of active caspase-3 was higher after VTo than in the CT ovaries. The pro- to anti-apoptotic mRNA expression ratio (*Bax/Bcl-xl*) increased in all groups after *in vitro* culture, confirming the previous TUNEL analysis result showing no effect on the apoptosis incidence immediately after the thawing of vitrified ovaries^49^. The fact that the *Bax/Bcl-xl* ratio of the VTo-cult ovaries dramatically increased compared to that of the SF-cult or fresh CT-cult ovaries could indicate that VT changes the susceptibility of the ovaries to apoptotic signals.

Moreover, the analysis of the mRNA levels of *Gdf9*, an oocyte-derived factor involved in folliculogenesis, showed that the levels were similar between all groups. On the other hand, the *Bmp15* and *Amh* mRNA levels were highly impacted by cryopreservation and culture, with a highly significant decrease in the mRNA levels of both genes after the *in vitro* culture of SF or VTo ovaries, as previously described^50^. A similar study with human ovarian tissue also found that the VT ovaries produced less AMH *in vitro* than the CT and SF ovaries^51^.

Because most cryopreservation-associated damage can only be detected later, the transplantation of SF or VT ovaries to restore the fertility of young mice with induced premature ovarian failure^34^ may be the best way to evaluate the developmental potential of the ovary after warming, as previously suggested^23^. In our study, we chose to use the chemically induced ovarian failure model to better represent the human clinical condition. Indeed, the injection of VCD directly targets the pool of primordial and primary follicles of the ovary. Moreover, VCD does not have toxic effects on non-ovarian tissues^52^. Here, unilateral ovariectomy was performed before VCD injection, allowing orthotopic auto-transplantation in the follicle-depleted ovary. This model offered the advantage of mimicking the clinical situation for women facing premature ovarian failure after the treatment of their cancers and for whom ovarian tissue cryopreservation was performed before therapy.

There is no consensus on the optimal VT protocol, and in our *in vitro* study, only one protocol and system were chosen^30^. Notably, from a physical point of view, VT performs with the open system with a direct exposure to liquid nitrogen in a virtually absent volume of medium represents the ideal procedure^53^. However, the risk of contamination with pathogens during the VT procedure or cross-contamination during long-term storage with open system may increase. To overcome such risks, several closed systems have been developed. Because of the thermal insulation of samples in closed VT devices, a decreased cooling rate relative to that of open VT systems could lead to ice crystal formation, resulting in cell death or reduced developmental capacity. One limit of our study is, that we used the same cryopreservation medium for both VT procedures (open and closed). Furthermore, the warming procedure was performed at 37 °C for both VT procedures. The first step of cryoprotectant unloading was performed at 37 °C for both VT methods as described by others^32,54–56^. Since there is no consensus for tissue warming after VT, the next steps were also performed at 37 °C. Anyway, to further improve the ovarian VT protocol, warming could be done at room temperature.

In the second part of this study, the *in vivo* results showed that after auto-transplantation with all types of cryopreserved ovaries (SF, VTo or VTc), most of the mice recovered their oestrous cycles. However, only those transplanted with SF or VTo ovaries became pregnant during the next 20 weeks after grafting. Moreover, the litter size tended to be higher in the SF-transplanted group than in the VTo group (3.7 ± 0.51 versus 2.8 ± 0.86). Our results partially confirm those of Chen *et al*., who developed a VT method followed by allogeneic orthotopic transplantation^57^. One striking difference with this study resides in the composition of cryopreservation media. Some live births have also been described after the VT and orthotopic allografting of 10-day-old green fluorescent protein (GFP)-transgenic mouse ovaries^58^. However, the number of GFP-positive pups born by natural mating was lower in the graft ovary group than in the CT group, leading to similar results to our own.

We also noticed that the number of surviving litters was different between the mice grafted with SF ovaries and those grafted with VTo ovaries (47% versus 12.5%). In conventional breeding, the pup mortality rates of laboratory mice are commonly high in the first litter, and the inability of primiparous females to care appropriately for their offspring has been reported^59^. The pup mortality rate of a litter depends on the mouse strain, with C57Bl/6 mothers having significantly higher pup mortality than DBA/2 J mothers, with more pup deaths in the first than in the second litter and with most deaths occurring within the first 3 days after parturition^60^.

Histological analyses of ovaries that were recovered 8 months after grafting showed that SF ovaries from mice that were previously pregnant displayed similar follicular development and corpora lutea formation to the CT ovaries, whereas the both VTo ovaries and SF ovaries recovered from the mice that were never pregnant displayed only a few corpora lutea and no more primordial or growing follicles.

## Conclusion

Our *in vitro* results indicate that differences between SF and VTo ovaries immediately after thawing/warming are not evident by histo-morphological analysis of cryopreserved ovarian tissue. However, immunohistochemical and RT-qPCR analyses for apoptosis and specific follicular proteins are altered after culture, mostly in VTo ovaries compared to SF ovaries. In addition, auto-transplantation of SF ovaries, as well as VTo ovaries, fully restores mouse fertility and achieved the highest number of pregnancies. Even though auto-transplantation with VTc ovaries restored ovarian cycles, no pregnancies were achieved.

## Supplementary information

Supplementary information
