# Supplementary material for: Slow Freezing Versus Vitrification of Mouse Ovaries: from Ex Vivo Analyses to Successful Pregnancies after Auto-Transplantation
Source: Sci Rep. 2019 Dec 23;9:19668. doi: 10.1038/s41598-019-56182-8 (PMC6928220; doi:10.1038/s41598-019-56182-8)

SLOW FREEZING VERSUS VITRIFICATION OF MOUSE OVARIES: FROM *EX VIVO* ANALYSES TO SUCCESSFUL PREGNANCIES AFTER AUTO-TRANSPLANTATION

Carmen Terren, Maïté Fransolet, Marie Ancion, Michelle Nisolle & Carine Munaut

Table S1: Experimental distribution of ex vivo analysed ovaries

**Analysis**	**CT**	**CT cult**	**SF**	**SF cult**	**VT**	**VT cult**
**IHC**	16	10	5	5	4	5
**RTqPCR**	22	10	9	9	9	9
**WB**	16	10	7	5	6	6
**Total**	54	30	21	19	19	20

Table S2: p-values associated to individual differences between the experimental groups corrected for multiple testing (Bonferroni's correction)

**Comparisons**	**Pregnant mice**	**Number of pregnancies**
	p-values	p-values
**CT versus SF**	ns	ns
**vs VTo**	ns	0.4632
**vs VTc**	**0.0008 (***)**	**0.0044 (*)**
**SF versus VTo**	ns	0.1472
**vs VTc**	**0.0008 (***)**	**0.0132 (*)**
**VTo versus VTc**	**0.0156 (*)**	NA

NA : not applicable ; ns : non signoificant

Figure S1: Representative western blot for (a) caspase-3, (b) LC3 and (c) BMP15 proteins. CT: control/fresh ovaries; SF: slow frozen/thawed ovaries, VTo: open vitrified/warmed ovaries, cult: *ex vivo* culture during 4 hours.

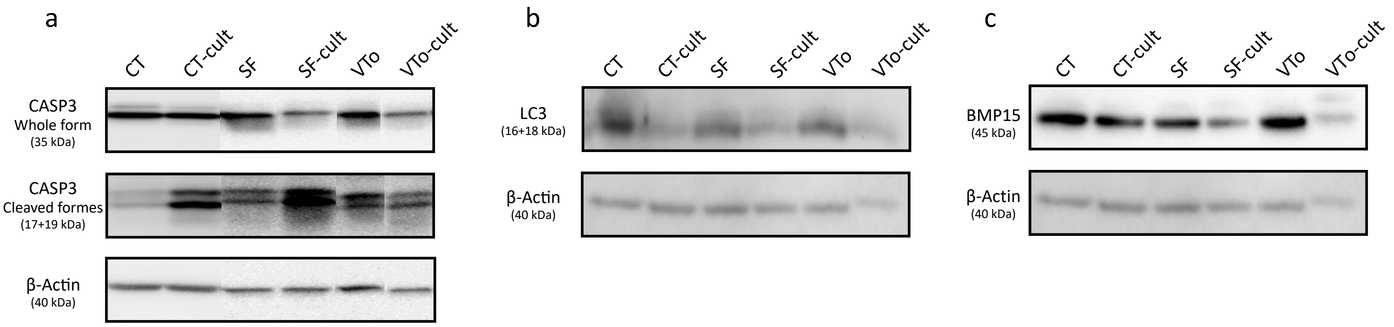

Figure S2: Experimental design and time line for the mice model of induced ovarian failure.

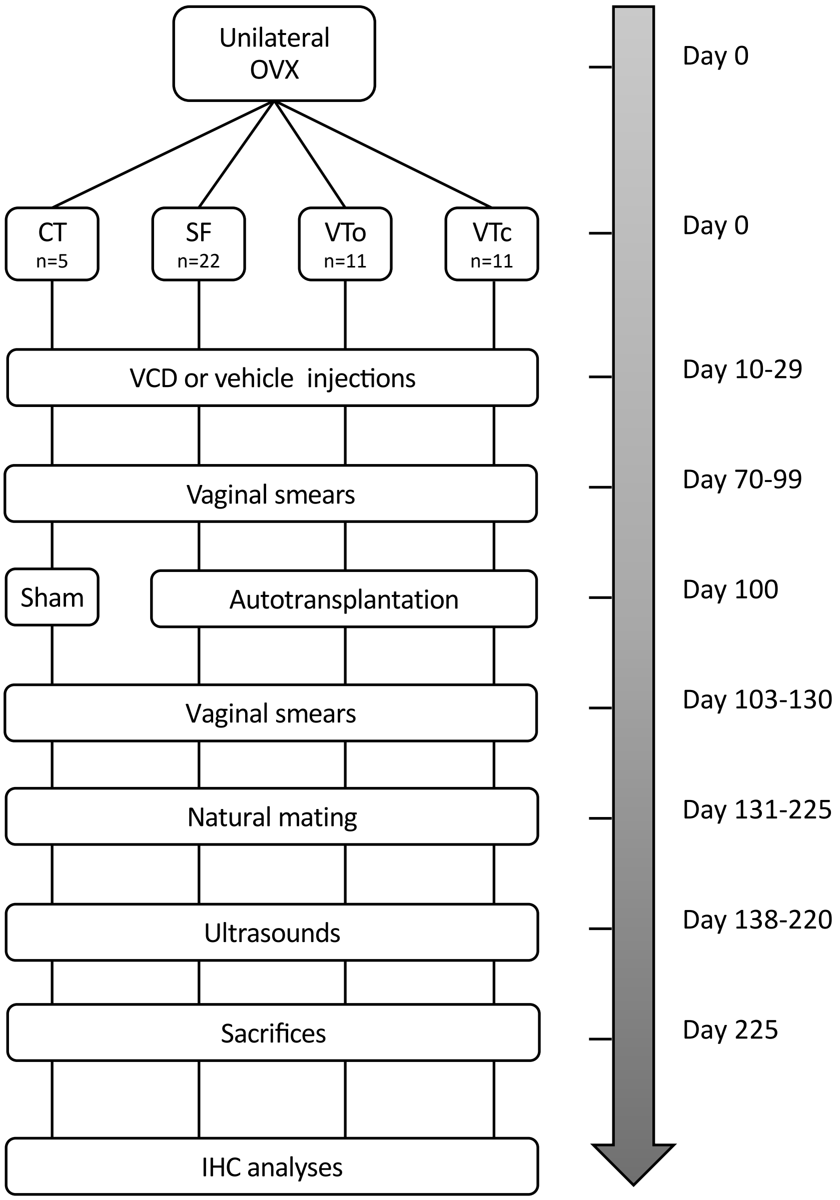

Figure S3: Follow-up of the induction of menopause by daily vaginal smears from 8 to 11 weeks after the first injection of VCD in unilaterally ovariectomized mice. n=44 mice

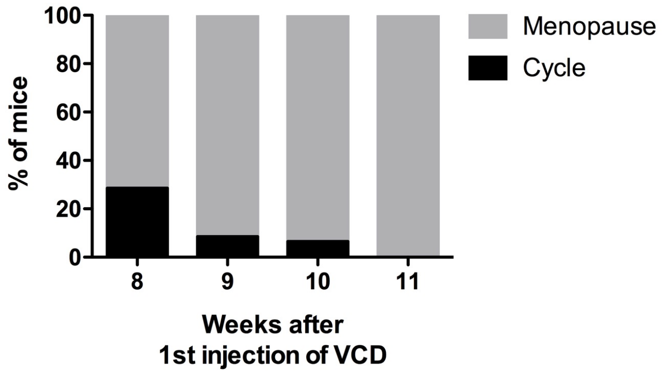

Supplement: Supplementary file 1 — Supplementary information [file 41598_2019_56182_MOESM1_ESM.pdf]
